# Peer support in acute psychiatric inpatient settings: A scoping review

**DOI:** 10.1002/pcn5.70248

**Published:** 2025-11-14

**Authors:** Michio Maruta, Gwanghee Han, Kikuo Eguchi, Daiki Yamazono, Tomofusa Kawano, Yuri Onomichi, Miki Yamashita, Toshio Higashi, Akira Imamura, Suguru Shimokihara, Goro Tanaka

**Affiliations:** ^1^ Graduate School of Biomedical Sciences Nagasaki University Nagasaki Japan; ^2^ Department of Occupational Therapy, School of Health Sciences at Fukuoka International University of Health and Welfare Okawa Japan; ^3^ Department of Occupational Therapy, Faculty of Rehabilitation Reiwa Health Sciences University Fukuoka Japan; ^4^ NPO Nagasaki Nozomi‐kai Nagasaki Japan

**Keywords:** inpatients, mental disorders, mental health recovery, peer group, review literature

## Abstract

Peer support, grounded in lived experiences, plays an important role in advancing recovery‐oriented mental healthcare. Although widely implemented in community settings, its adoption in acute psychiatric inpatient care remains limited because of structural and cultural factors that constrain recovery‐oriented practices. This scoping review aimed to map existing evidence on peer support in acute psychiatric inpatient settings, focusing on (1) implementation formats, (2) peer roles, and (3) facilitators and challenges. Following the Arksey and O'Malley framework, we conducted a scoping review using the participants, concepts, and context framework. We systematically searched the PubMed, Scopus, MEDLINE, CINAHL, and ProQuest databases. We included studies that involved individuals with mental health conditions, described peer support provided by persons with lived experiences, and were conducted in acute psychiatric inpatient settings. Eleven studies, published between 1995 and 2024, met the inclusion criteria. Peer support was reported as being implemented through informal and structured formats that vary in frequency, continuity, and delivery contexts. Peer roles included emotional, practical, advocacy, and relational support. The reported benefits include increased activity participation, emotional reassurance, social engagement, and recovery‐related gains for both patients and peer workers. Facilitators include training, supervision, leadership support, and collaborative relationships with staff. The reported challenges include unclear role boundaries, staff resistance, emotional strain, and structural limitations, including space, resources, and barriers to team integration. The findings suggest that peer support may help promote recovery‐oriented practices in acute psychiatric settings, although further rigorous studies are needed to confirm its impact and inform sustainable integration.

## INTRODUCTION

Recovery‐oriented practices have gained global prominence as a foundational paradigm in mental health care.^1^ The World Health Organization emphasizes prioritizing person‐centered, recovery‐oriented, and human rights‐based care to transform mental health systems.^2^ This approach focuses on fostering dignity, empowerment, and community integration.

Although widely adopted in community‐based services, recovery‐oriented principles remain less consistently implemented in inpatient settings, particularly in acute psychiatric wards.^3^ Structural constraints, such as short admissions, risk‐focused protocols, and limited opportunities for shared decision‐making, often hinder the application of such principles in these settings.^1^, ^4^, ^5^ These constraints can compromise key recovery domains such as hope, identity, and connectedness. Staff working in acute psychiatric settings have reported tensions between ideal recovery and institutional demands.^6^, ^7^ Likewise, service users hospitalized in these settings have also described feeling overlooked or misunderstood by staff, highlighting a lack of emotional safety and human connection.^8^ Despite these broader challenges, inpatient hospitalization offers a valuable opportunity to support individuals in initiating their recovery journey after discharge.^9^ This underscores the need to explore supportive approaches that can facilitate recovery, even within the constraints of acute psychiatric care.

Peer support has emerged as a promising strategy for advancing recovery‐oriented practice.^10^ A growing body of evidence from systematic reviews suggests that peer support positively influences recovery, hope, empowerment, and quality of life.^11^, ^12^, ^13^ Peer supporters are individuals with lived experiences of mental health challenges who offer support grounded in shared understanding, mutual respect, and empowerment. Their roles emphasize emotional connections and foster self‐determination and hope.^10^, ^14^ By sharing their recovery experiences, they help reduce stigma and build trust in ways that differ from traditional clinical roles.^14^, ^15^ In some studies, peer support has been described by service users as one of the most positive aspects of their hospitalization in acute psychiatric settings, and is sometimes perceived as more beneficial than professional care.^8^, ^16^, ^17^ Furthermore, Niimura et al.^18^ proposed that placing peer workers or others with similar lived experiences in acute inpatient wards could help provide timely guidance rooted in a shared understanding. Peer support may act as a bridge between inpatient care and community reintegration, offering the continuity of recovery through shared experience.^18^

Despite growing recognition of the value of peer support, evidence regarding its implementation and impact in acute psychiatric inpatient settings remains limited and fragmented. To our knowledge, no study has comprehensively synthesized how peer support is delivered, the roles peers play, and the challenges and outcomes associated with their involvement in high‐intensity environments. To address this gap, the present scoping review systematically mapped the existing literature on peer support in acute psychiatric inpatient settings. It aimed to clarify (1) how peer support is implemented in practice; (2) the roles peer supporters play in these settings; and (3) the facilitators and challenges associated with its implementation.

## METHODS

This scoping review followed the Arksey and O'Malley methodological framework,^19^ refined by Levac et al.,^20^ and aligned with the latest guidelines of the Joanna Briggs Institute (JBI).^21^ We applied six steps: (1) identifying the research question; (2) identifying relevant studies; (3) study selection; (4) charting the data; (5) collating, summarizing, and reporting the results; and (6) consulting the stakeholders. We also followed the Preferred Reporting Items for Systematic Reviews and Meta‐Analyses extension for Scoping Reviews checklists.^22^ This review protocol was not preregistered.

### Step 1: Identifying research question

We conducted a preliminary literature review and identified key gaps in the literature. While peer support is increasingly recognized in mental health systems, no previous review has systematically examined how it is implemented, specifically in acute psychiatric inpatient settings. Given the unique challenges and dynamics of acute care environments, understanding the practical implementation of peer support in these settings is essential to inform service development and enhance recovery‐oriented practices. To clarify the scope and focus of this review, we developed the following research questions following the Participants, Concept, and Context (PCC) framework.^21^ The following research questions guided this review:

1. How is peer support implemented in acute psychiatric inpatient settings?
2. What roles do peers play in these settings?
3. What are the facilitators and challenges associated with the implementation of peer support in acute psychiatric inpatient care?

### Step 2: Identifying relevant studies

In October 2024, we conducted a preliminary search using terms related to “peer support, psychiatric care, inpatient care,” and “acute and subacute settings.” The search strategy was refined using previous studies to ensure comprehensive coverage of peer support for mental health.^23^, ^24^ The final search was conducted on January 9, 2025, using PubMed, Scopus, MEDLINE, CINAHL, and ProQuest databases. The detailed search strategies are provided in Table S1.

### Step 3: Study selection

Based on the PCC framework (Table 1), we included studies that involved individuals hospitalized in psychiatric inpatient settings (participants), described the implementation of peer support delivered by individuals with lived experiences of mental health challenges (concept), and were conducted within acute psychiatric inpatient settings (context).

**Table 1:** Identifying the research question and eligibility criteria.

	Inclusion criteria	Exclusion criteria
Participants	Individuals admitted to psychiatric inpatient settings	None specified; exclusions based on context and concept
Concept	Studies describing the implementation of peer support provided by individuals with lived experience of mental health challenges	Studies that did not include peer support provided by individuals with lived experience Studies in which peer support was not directed toward patients or their families
Context	Studies conducted in acute or subacute psychiatric inpatient settings	Studies conducted outside psychiatric inpatient settings Studies in which peer support was delivered during hospitalization, but primarily focused on post‐discharge or community transition support
Article type	Original research articles (qualitative, quantitative, mixed‐methods, case studies, pilot studies)	Non‐research publications (e.g., reviews, editorials, commentaries, protocols without results) Articles not published in English

We excluded studies that did not include peer support provided by individuals with lived experiences, were not conducted in psychiatric inpatient settings, focused on peer support directed at individuals other than patients or their families, were not original research articles, or were not available in English. In addition, studies in which peer support was delivered during hospitalization, but primarily focused on post‐discharge or community transition support, were excluded.

Following the systematic searches, all identified citations were uploaded to Rayyan (https://www.rayyan.ai/) and duplicates were removed. The titles and abstracts were independently screened by the first author and four other contributors. Full texts were reviewed if the abstracts lacked the exclusionary information. Disagreements were resolved through discussions among all five authors who contributed to the study.

### Step 4: Charting the data

A data‐charting form was developed using Microsoft Excel to systematically extract and organize relevant information from the included studies. The first author created the initial version of the form based on the research questions and inclusion criteria, which was reviewed by other authors to ensure comprehensiveness and clarity. Data were extracted by the first author and subsequently reviewed and verified by the other authors. This approach is consistent with the current scoping review guidelines, which permit single‐reviewer extraction with verification, provided that the process is transparently reported.

We charted the basic study characteristics, including author (s), year, country, study aim and design, setting, participants, length of hospital stay, diagnostic information of peer supporters and patients, and key findings. To clarify how peer support is implemented in acute psychiatric inpatient settings, we extracted and organized information on the support format, frequency, and continuity, context of support, employment status of peers, roles of peers, and factors that facilitate or challenge implementation. Where studies did not explicitly identify facilitators or challenges, we extracted relevant information based on the descriptions in the Methods, Results, and Discussion sections. Following the guidance of the JBI Manual for Evidence Synthesis,^21^ this scoping review did not include a formal risk of bias assessment. The primary aim was to map and describe the available evidence, rather than to evaluate the effectiveness of interventions.

### Step 5: Collating, summarizing, and reporting the results

The extracted data were summarized descriptively and organized into summary tables to present the key study characteristics and details related to the implementation of peer support in acute psychiatric inpatient settings. These tables form the basis for the narrative synthesis and address the review questions.

### Step 6: Consultation with stakeholders

To enhance the practical relevance and interpretive validity of our findings, a brief consultation was conducted with two peer supporters and one occupational therapist actively engaged in peer support practice. The occupational therapist is employed full‐time, and the two peer supporters work part‐time at a Community Activity Support Center established under the Comprehensive Support for Persons with Disabilities Act in Japan. Within this center, peer support is integrated into daily care. Both peer supporters also have personal lived experience of admission to an acute psychiatric ward. One had been occasionally dispatched from the Community Activity Support Center to provide peer support to patients in acute psychiatric wards. The other does not currently work directly in acute wards but has extensive experience in diverse peer support practices and is actively involved in discussions concerning acute inpatient care.

A summary of the preliminary findings and three open‐ended questions were shared with the co‐authors. Feedback was obtained through group discussions involving all three co‐authors. This process was not a separate data collection phase but a collaborative step to validate the findings, identify overlooked perspectives, and enhance practical relevance. These insights have been incorporated into the Discussion section.

## RESULTS

### Study characteristics

**Figure 1:**
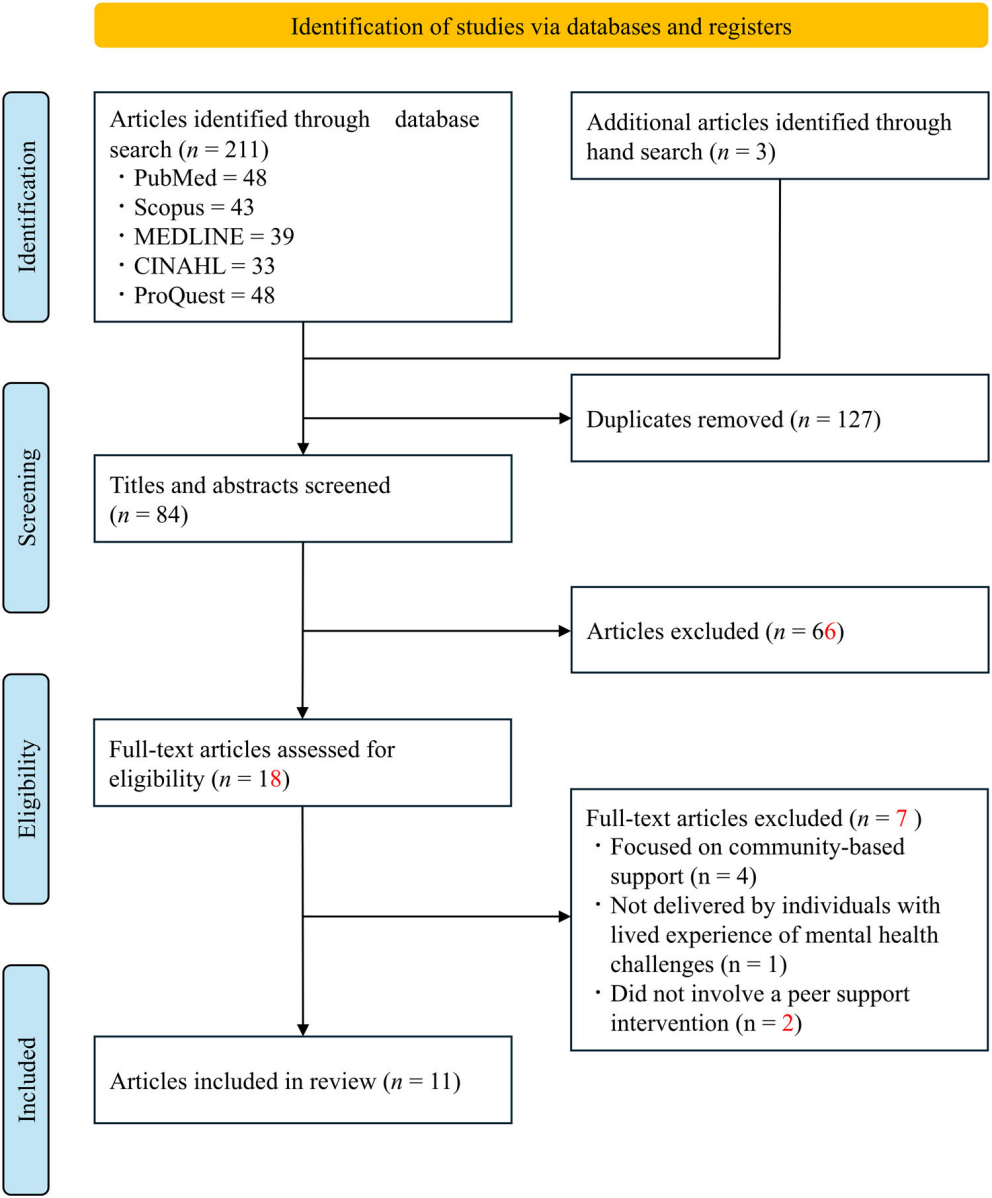
Preferred reporting items for systematic reviews and meta‐analyses flowchart for the scoping review.

Of the 84 identified studies, 11 met the inclusion criteria. Figure 1 illustrates the study selection process. At the title and abstract screening stage, records were excluded for the following reasons: absence of peer support provided by persons with lived experience (*n* = 42), studies not conducted in psychiatric inpatient settings (*n* = 2), focus on peer support for populations other than patients or their families (*n* = 3), non‐original research (*n* = 15), and focus on post‐discharge or community transition support (*n* = 4). Table 2 presents an overview of the included studies. We included studies conducted in the United States, Canada, the United Kingdom, Italy, Germany, and New Zealand, and published between 1995 and 2024. Most studies employed qualitative designs (*n* = 5), with additional studies utilizing qualitative–quantitative approaches (*n* = 2), program descriptions (*n* = 2), case studies (*n* = 1), and controlled pre‐post designs. The majority were conducted in acute psychiatric inpatient settings, whereas others focused on psychiatric emergency departments, highly secure forensic units, or intensive mental healthcare environments. The study participants included adult inpatients, peer support workers (volunteers or employed), and, in some cases, staff members. Three studies examined naturally occurring peer support that emerged within inpatient settings without formal programmatic structures, whereas the remaining eight studies focused on structured or intentional peer support interventions.

**Table 2:** Characteristics of included studies.

Authors/Year Country	Aim	Design	Setting	Participants	Diagnoses/Conditions	Length of stay	Key Findings
Franco et al.^28^ (USA)	To foster voluntary, active patient participation in simultaneous mental illness and addiction recovery and network patients with community resources, utilizing a combined token economy and self‐help model.	Program description and evaluation study	Acute inpatient psychiatric ward	Over 1000 dually diagnosed inpatients	Patients are simultaneously diagnosed with psychiatric illness and substance abuse	Average 1 month	The peer‐led token economy enhanced participation in activities, reduced violence on the ward, improved the daily routine, and increased patient self‐esteem and preparation for community living.
Bouchard et al.^25^ Canada	To explore perceptions and experiences of naturally occurring peer support among adult mental health inpatients.	Qualitative descriptive study	Adult mental health inpatient units	Ten adult mental health inpatients	Depression, schizophrenia, nonspecific psychotic disorders	10 to 602 days (mean 137.7, median 72)	Peer support is extensive, beneficial, and occurs spontaneously among inpatients, independent of staff involvement. It encompasses diverse helping actions (e.g., material, ADL, emotional, and advice). This leads to improved mental health outcomes and quality of life for both providers and recipients.
Stone et al.^35^ UK	To explore the experience of peer support workers in their new role and to examine the extent to which peer support contributes to recovery‐based practice within the NHS context.	Qualitative study	An acute psychiatric ward	Five peer support workers	No specific diagnosis was reported	Not reported	Peer support workers reported personal growth, improved confidence, and recovery benefits. Their initial challenges with staff acceptance were overcome over time. The role also promoted inclusion, hope, and supportive relationships. Additionally, safety concerns on the ward were noted.
Migdole et al.^29^ USA	To describe the development and implementation of a recovery‐oriented peer support team in a psychiatric emergency department and explore its benefits and challenges.	Program descriptive study	Psychiatric emergency department	Patients in the psychiatric emergency department	No specific diagnosis was reported	Not reported	Greater mean satisfaction among emergency department consumers when peer staff were present. Provided meaningful, competitive employment for peers, increasing confidence. Peers educated medical students on recovery concepts. Challenges included peer absenteeism and related issues, trauma triggers for peers, and apprehension among staff.
Smith et al.^31^ USA	To clarify the potential role and impact of behavioral health peer support providers on community hospital acute inpatient psychiatric units.	Qualitative study	Community hospital acute inpatient psychiatric units	14 participants: 6 behavioral health peers; 8 recipients of peer services	Patients: No specific diagnosis was reported. Peer supporters: No specific diagnosis was reported (bipolar illness mentioned as an example).	Not reported	Positive initial reactions and strong emotional connections to peers were observed. Peers supplemented clinical care in engagement, discharge planning, and care transitions. Challenges included clinical team interactions and blurred professional boundaries.
Wolfendale & Musaabi^30^ UK	Provide an overview of the implementation of a peer support volunteer scheme in a high secure setting. Explore the experiences of peer support volunteers conducting this role, based predominantly on an assertive rehabilitation ward.	Case study	A high secure forensic mental health service	One peer support volunteer; eight newly transferred service users	No specific diagnosis has been reported (the hospital primarily treats mental illness and personality disorders).	Not reported	The peer support volunteer scheme facilitated patient adjustment, increased social engagement, and reduced anxiety in a high secure forensic mental health setting. Peer supporters gained confidence and skills while also easing staff workload. Challenges included boundary establishment and power imbalances, especially in forensic settings.
Galloway & Pistrang^27^ UK	To provide a detailed picture of service‐users' experiences of giving and receiving naturally occurring peer support and challenges in an acute inpatient setting; obtain staff perceptions.	Qualitative study	Five single‐sex adult acute inpatient units	12 service‐user; seven staff	Mood disorder, Psychosis, Personality disorder	5 to 6 weeks	Service‐users described highly valued supportive interactions and identified challenges/barriers. Staff accounts were broadly consistent with service users' themes; however, they emphasized potential risks and focused on the functional value of peer support in facilitating treatment engagement, ultimately showing a more limited understanding of its nature and process. Providing support was meaningful to service‐users, promoting agency.
Cooper et al.^26^ New Zealand	To explore service user experiences of naturally occurring peer support on the acute mental health unit.	Qualitative study	Four acute mental health units	43 service users	No specific diagnosis was reported.	Not reported	Naturally occurring peer support occurred through three themes: fulfilling a need to connect, desire to improve the unit experience for others, and a sense of solidarity among service users.
Badouin et al.^33^ Germany	To assess the effectiveness of employing peer support workers with regard to reducing the use of restraints.	Prospective controlled pre‐post study	Two locked acute psychiatric wards	923 patients	Patients: F01, F1X, F2X, F3X, F4X, F5X, F6X, F7X, F8X, Non‐psychiatric main disorder* Peer supporters: No specific diagnosis was reported.	Not reported	Primary outcome: restraint use (proportion and frequency per patient). Restraint use increased in the control ward but remained stable in the peer‐support ward, with no change in restraint duration in either group, suggesting a potential protective effect of peer support.
Di Lorenzo et al.^32^ Italy	To evaluate group psychotherapy sessions implemented in an acute psychiatric ward by analyzing the group type according to Bion's basic assumptions, the main narrative themes, the mentalization processes, the participants' adherence, and the role of peer support providers within this setting.	Qualitative–quantitative retrospective study	Acute psychiatric ward	239 inpatients	Patients: Schizophrenia spectrum disorders, Bipolar disorders, Personality disorders, Intellectual disability, Organic psychotic conditions, Dysthymia and depressive disorders, Anorexia, and Others. Peer supporters: No specific diagnosis was reported.	11.2 ± 2.1 days	Primary outcome not explicitly stated; the main quantitative finding was a significant correlation between the Mentalization‐Based Therapy Group Adherence and Quality Scale overall occurrence score and participation variables: a positive association with the number of group participants (Coef. = 14.87) and a negative association with the number of active speakers (Coef. = −16.87). Qualitative analyses suggested that the regular presence of the peer support provider fostered group continuity and cohesion, maintained group memory, and helped patients reduce anxiety and helplessness.
McEwan McManus et al.^34^ UK	To develop and deliver a Hearing Voices Group to create a safe space for voice hearers to share experiences and access support, and to discuss feedback received.	Qualitative–quantitative descriptive study	Acute and intensive mental health care settings	32 service users	Patients: No specific diagnosis was reported (People who have experienced voices/auditory hallucinations or have unusual experiences). Peer supporter: Psychosis	Not reported	Primary outcomes: quantitative evaluation indicated high group effectiveness and acceptability, with most participants rating delivery, pace, and facilitation positively on a 5‐point Likert scale. Qualitative feedback supported these findings, describing the groups as therapeutic, validating, and enhanced by the presence of an expert by experience and staff collaboration.

Abbreviation: ADL, Activities of daily living

* Diagnostic categories were defined according to the International Classification of Diseases, 10th Revision (ICD‐10). Statistical analyses included participants with diagnoses corresponding to F1X, F2X, and F3X.

A summary of the peer support implementation, covering formats, roles, and influencing factors, is presented in Table 3.

**Table 3:** Implementation characteristics of peer support in included studies.

Authors	Support format	Frequency and continuity	Setting/Context of support	Employment status	Role of peer support	Facilitators of implementation	Challenges to implementation
Franco et al.^28^	Peer‐led group activities, peer rating of behavior, patient involvement in ward store operations, and new patient orientation.	Daily group meetings and peer support roles were sustained throughout hospitalization.	Conducted within the daily ward routine. Occurred during scheduled and patient‐initiated group discussions and peer rating of behaviors.	Peer inpatient	Peer leaders rated peers' behavior, delivered reinforcers in the ward store, organized and initiated group activities, and oriented new patients to ward rules and routines.	Training was standardized via videotapes, staff supervision, patient inclusion in ward management, and the combined use of self‐help and behavioral approaches.	Staff objection or resistance to acutely ill patients reliably observing peers.
Bouchard et al.^25^	Informal, naturally occurring, spontaneous, and mutual peer support among inpatients.	Naturally occurring	Broad contexts within inpatient settings, including daily routines and leisure activities.	Peer inpatient	Helping with activities of daily living, sharing material goods, providing information and advice, sharing a social life, and offering emotional support.	Peers' personal traits (e.g., natural helper, empathetic, common experiences), positive group dynamics (e.g., mutual respect, friendships), and staff introductions to new patients.	Peers' personal traits (e.g., inability to ask for help, withdrawn behaviors), negative group dynamics (e.g., not getting along), and staff practices (e.g., discouraging support, lack of recognition).
Stone et al.^35^	Formal intentional peer support.	Twice weekly to patients during their admission.	Provided within an acute psychiatric ward environment.	Employed	Help others to develop alternative narratives about their lives through specific communication. Provide a space for patients to talk. Contribute to recovery‐based practice.	Adequate supervision and ongoing support from the mental health charity and supervisor, positive training, including group discussion, supportive peer group among PSWs, and friendly and helpful ward staff relations.	Initial challenges involved staff acceptance and working relationships, safety concerns arising from patient violence and aggression on the ward, and coping with a distressing work environment.
Migdole et al.^29^	Informal, one‐to‐one support.	Available during shifts. Peer staff generally worked in pairs, rotating shifts every 2 h.	Provided within the locked crisis intervention unit, specifically in patient lounge areas, patient rooms, or the nursing area.	Employed	Orienting patients to emergency department policies and their rights, providing emotional support, facilitating communication, sharing recovery stories, educating medical students on recovery concepts, and challenging stigma.	Strong leadership from the medical director and an APRN champion, comprehensive peer training, accessible supervision, fostering integration with existing staff, and a positive hospital relationship.	Peer absenteeism, professional conduct issues, trauma triggers for peers, peers needing emergency department services, staff apprehension about working with peers, and limited physical space.
Smith et al.^31^	No standard service format.	No standard frequency and continuity.	Daily routines, discharge/care transitions, and practical support in community settings	Employed	Peer supporters engaged recipients, providing empathy validation, hope, and practical support, including discharge planning and community accompaniment. They also shared lived experiences, advocated for needs, and conducted groups in the hospital.	Formal training (engagement, advocacy, wellness); peer competence, professionalism, hope‐inspiring roles, and use of shared experience for connection	Interactions/obstacles with clinical treatment team, blurred professional‐friendship boundaries, structural limitations of peer relationships, and individual conflicts with peers.
Wolfendale & Musaabi,^30^	Structured one‐to‐one formal induction for new patients.	Induction within 24 h; initial shadowing by peer supporter; ongoing support levels documented per patient.	Primarily ward‐based; includes orientation of new patients, introductions to peers and staff, and a scheduled time to complete the induction checklist together.	Volunteer (trained)	Orientating new patients to the environment, introducing them to peers and staff, providing formal induction, helping patients learn ward atmosphere, shadowing, and co‐producing information booklets.	Full training program, daily staff support, negotiated formal supervision, multi‐disciplinary team and ward manager support, inclusive scheme development, and external endorsement.	Establishing appropriate boundaries; dilution of the role due to power imbalances (between volunteer/recipient and clinical team/scheme).
Galloway & Pistrang,^27^	Informal, naturally occurring, spontaneous, and mutual peer support among inpatients.	Naturally occurring	Broad inpatient contexts, including mealtimes, common rooms, and bedrooms, involved greetings, sharing items, and conversations.	Peer inpatient	Companionship and friendship, responding to distress and talking about personal issues (listening, offering perspective), encouragement, mutual learning, and helping navigate the ward.	Service‐users' thoughtfulness and responsiveness to emotional needs; providing space to be upset, actively listening, talking openly about personal issues, knowing when to pull back, and offering encouragement.	Challenges included ward context (e.g., aggression, staff discouragement), “treading carefully” (e.g., fear of disclosure, reactions). Also, personal difficulties (e.g., illness, trust issues, distress from helping others) and staff emphasis on risks.
Cooper et al.^26^	Informal, naturally occurring, spontaneous, and mutual peer support among inpatients.	Support occurred irregularly and when needed or when opportunities arose. It was not scheduled or formalized.	Broad contexts within inpatient settings, including everyday life on the unit.	Peer inpatient	Providing social connection and understanding, supporting adjustment and recovery, offering acts of kindness, instigating activities, and fostering a sense of solidarity among inpatients.	Peer support was driven by service users' unmet need for connection, desire to improve others' unit experience, and a sense of solidarity from shared experiences.	Challenges included staff failing to recognize or actively discouraging it, a lack of social interaction opportunities due to unit design and resources, and emotional risks for service users providing support without backup.
Badouin et al.^33^	Peer support workers engaged in individual and group settings with patients.	7 h/week per PSW; 22‐month period with 5‐month reduction due to early departures.	Peer support was implemented in one locked ward. It took place in clinical and outpatient settings, assisting home treatment teams or joining patients in their private environments.	Employed	Peer support workers initiated direct and personal contact with patients, engaged in individual/group activities, trained staff on recovery‐focused matters, exchanged experiences with practitioners, and approached decision‐makers to facilitate patient recovery.	Implementation was aided by senior management support, a team‐building day to address challenges, and biweekly supervision from mentors. Recovery‐oriented on‐the‐job training was also provided.	Major challenges included the COVID‐19 pandemic, staff reorganization, initial role uncertainty, early peer support worker departures (causing a 5‐month gap), and a change of consulting psychiatrist.
Di Lorenzo et al.^32^	Integrated within structured group psychotherapy sessions.	Weekly sessions (45 min each).	Conducted within structured group therapy.	Not reported (Unpaid)	Maintained group memory, acted as glue with the outside, offered an empathic identification model, and shared illness experience to help reduce anxiety and helplessness.	Regular presence of a peer support provider.	Not reported
McEwan McManus et al.^34^	Peer‐led, structured group sessions.	Each group session ran for 30–45 min	Conducted within structured group therapy.	Employed (Assistant Psychologist with expert by experience)	Creating a safe space for voice hearers to share experiences and access support, sharing lived experience and coping skills, and fostering validation and connections.	Staff and ward‐based promotion were paramount; 1‐1 nurse engagement helped recruitment; close collaboration with nursing staff ensured group cohesion.	Logistical issues (facilitator illness/unforeseen circumstances).

### Overview of reported benefits and key findings

Informal and spontaneous peer support has been described as widespread and emotionally meaningful, involving material aid, emotional support, and advice for inpatients.^25^ Such support was reported to emerge through everyday connections, efforts to improve the ward experience for others, and a shared sense of solidarity among patients.^26^ Providing support was described as meaningful by the service users, although challenges and barriers were also noted.^27^

Structured peer support has been reported to increase participation in activities, reduce ward violence, improve daily routines, and enhance self‐esteem.^28^ Other studies have described greater satisfaction with care during periods of peer staff involvement,^29^ support for patient adjustment and social engagement,^30^ and assistance with clinical care in engagement, discharge planning, and care transitions.^31^ Peer support has also been described as helping to sustain group continuity and reducing anxiety and helplessness,^32^ especially in settings where no increase in the use of restraints was observed.^33^ Participants in the peer‐led groups noted the value of shared experiences and coping strategies.^34^

Reported benefits for peer supporters themselves included increased confidence, personal growth, and recovery‐related gains.^30^, ^35^ However, reported challenges included role boundaries, staff apprehension, absenteeism, trauma‐related issues, and safety concerns.^29^, ^30^, ^31^, ^35^

### Implementation of peer support in acute psychiatric inpatient settings

Peer support has been implemented in both informal and structured formats. Informal support, described as spontaneous and mutually provided among inpatients, was reported in three of the eleven included studies.^25^, ^26^, ^27^ Structured formats include peer‐led group sessions, one‐to‐one support, formal orientation, and integration into therapeutic programs.^28^, ^30^, ^32^, ^34^, ^35^ For instance, in an Italian acute psychiatric ward, peer supporters were integrated into structured group therapy sessions, co‐facilitating discussions with clinicians and sharing recovery narratives that encouraged patients to reflect on life after discharge and fostered engagement.^32^ In another program in Germany, peer supporters were incorporated into a restraint reduction initiative, where they engaged in structured one‐to‐one conversations with patients, exchanged perspectives with clinical staff within the institutional system, and advocated to decision‐makers to promote recovery and empowerment.^33^

The frequency and continuity of peer support varied widely. Informal support was irregularly provided as required. Structured interventions ranged from daily^28^ or twice‐weekly^35^ sessions to weekly sessions.^32^ Peer workers were scheduled to rotate shifts in one study^29^ whereas others provided frequent and continuous support throughout hospitalization without a fixed schedule.^31^ In one study, peer workers engaged approximately 7 h/week over an extended implementation period.^33^

Support was provided primarily in inpatient wards, including lounges, bedrooms, and activity rooms, and was often embedded in daily routines or group settings. Some programs have been extended to community contexts or have included interviews and care transitions.^31^, ^33^

The employment status varied. Informal peer support was provided by inpatients.^25^, ^26^, ^27^ Structured programs employ peer workers,^29^, ^31^, ^33^, ^34^, ^35^ or rely on trained volunteers.^30^

### Roles of peers in acute psychiatric care

Peer supporters in acute psychiatric inpatient settings engage in various roles that encompass emotional, practical, advocacy, and relational support. Emotional support is central and includes listening, sharing lived experiences, offering encouragement, and providing space for emotional expression in informal or peer‐based interactions.^25^, ^26^, ^27^

Practical roles involved assisting with ward orientation, accompanying patients to scheduled activities, and supporting engagement in routines or discharge planning.^28^, ^31^ Peer supporters also participated in group interventions by co‐facilitating discussions and modeling recovery strategies.^32^, ^34^

Advocacy‐related roles include helping patients articulate concerns, navigate procedures, and advocate for their needs. It also involves educating staff and students on recovery‐oriented concepts and challenging stigma in clinical settings.^29^, ^31^, ^33^, ^35^

Additional roles included maintaining group memory, supporting adjustment and recovery, offering acts of kindness, instigating social activities, fostering a sense of solidarity, and strengthening social connections to reduce isolation and enhance ward cohesion.^26^, ^30^, ^32^, ^33^

### Facilitators and challenges in implementing peer support

Facilitators of peer support implementation include personal attributes of peers, such as empathy, shared experience, and helping orientation.^25^ Positive group dynamics and mutual respect also facilitate informal peer support.^25^ Formal training programs and supervision have been reported as key facilitators in several studies.^28^, ^29^, ^30^, ^31^, ^35^ All structured programs provided peer supporters with preparatory training and regular supervision or support. Training and supervision were typically delivered by psychologists, nurses, occupational therapists, or experienced peer workers. In several programs, team‐building activities and preparatory meetings were conducted prior to implementation to facilitate collaboration and clarify peer roles within the ward.^29^, ^33^ At the organizational level, factors such as strong leadership, inclusive development, and senior management support also aid implementation.^29^, ^33^ Further integration is supported by team communication, staff encouragement, and nurse collaboration,^30^, ^34^ and supportive factors such as friendly and helpful ward staff members,^35^ as well as a positive relationship with hospital personnel^29^ also strengthen peer support practices.

Challenges to implementation include staff resistance or lack of acceptance,^26^, ^27^, ^28^, ^29^, ^35^ uncertainty about peer roles,^33^ and staff practices that discourage peer interaction or support.^25^, ^26^, ^27^ Challenges related to peers included absenteeism, professional conduct issues, trauma triggers, and emotional strain.^29^, ^35^ Difficulties in managing boundaries have also been reported, including role confusion and power imbalances.^30^, ^31^ Additionally, structural and relational challenges include limited physical space, difficulties working within a clinical team, and conflicts among peers.^29^, ^31^ Contextual challenges included limited opportunities for social interaction due to unit design and resources, and logistical issues such as pandemic‐related disruptions and staff absences.^26^, ^33^, ^34^

## DISCUSSION

This scoping review mapped the existing evidence on peer support in acute psychiatric inpatient settings. Peer support was implemented in both informal and structured formats, with peers providing emotional, practical, advocacy, and relational support. Its implementation is influenced by staff attitudes, training, and institutional culture. Although peer support demonstrated potential benefits for both service users and peer supporters, its integration into acute settings varied across contexts and required thoughtful adaptation.

### How is peer support implemented in acute psychiatric inpatient settings?

Peer support has been implemented in both informal and structured formats in acute psychiatric settings. Informal support emerged spontaneously among the inpatients and was grounded in shared emotional experiences and mutual aid. These interactions reflect the core principle of mutuality, which refers to reciprocal and non‐hierarchical relationships based on shared experiences. This concept is central to peer support^36^, ^37^; its emergence, even in the absence of formal programs, highlights the latent capacity for resilience and human connection within restrictive institutional environments.^25^, ^26^, ^27^ In contrast, structured peer support involves trained and supervised peer workers integrated into clinical teams with clearly defined goals such as enhancing patient engagement or supporting discharge.^28^, ^29^, ^30^, ^31^ Implementation varied in intensity, continuity, and integration, reflecting factors such as organizational readiness, staff attitudes, and employment models. This diversity may reflect evolving approaches to integrating lived experiences with professional expertise rather than indicating a hierarchy of effectiveness.^38^, ^39^

Although peer support is adaptable to diverse clinical environments, its quality and sustainability in acute settings may vary depending on ward culture, institutional priorities, and patient acuity, particularly when it is not fully institutionalized. Therefore, rather than applying uniform models across settings, peer support is considered more effective when thoughtfully adapted to specific clinical and organizational contexts.

### What roles do peers play in these settings?

Peer supporters in acute psychiatric settings perform roles that extend beyond emotional support, including practical assistance, advocacy, and symbolic modeling. Their non‐judgmental presence and shared experiences create opportunities for connection and validation, which are elements often lacking in conventional inpatient care focused on risk management and clinical stabilization.^40^ Inpatients in acute psychiatric wards frequently report a sense of disconnection from everyday life as well as feelings of isolation and disorientation during hospitalization.^5^ By offering relational continuity and modeling recovery, peer supporters can help re‐establish a sense of coherence, restore a narrative of self, and foster belonging during this dislocating experience. Peer‐led interactions may foster psychological safety and hope during the early stages of recovery. Practical and advocacy functions, such as helping patients navigate routines or voice their preferences, may contribute to enhancing patients' sense of urgency and engagement with the institution.^41^, ^42^ Such roles may counterbalance the limited autonomy often experienced in acute psychiatric care and reinforce the distinct value of peer involvement.

The diverse peer roles may reflect the growing specialization and differentiation of peer support functions.^43^ However, the expansion of peer roles may be accompanied by concerns regarding role overload and ambiguity.^44^ In environments where relational boundaries are tightly regulated, attention to role clarity, interdisciplinary collaboration, and organizational support may be important for sustaining safe and effective peer involvement.^45^, ^46^

### What are the facilitators and challenges associated with the implementation of peer support?

This review identified a range of individual, interpersonal, and organizational factors that influence the implementation of peer support in acute psychiatric settings. Many structured peer support programs included in this review incorporated peer‐specific training and supervision. Prior studies suggest that such support reduces role ambiguity and anxiety among peer workers^47^ while also fostering a more peer‐supportive organizational climate and increasing the value placed on their role within organizations.^48^ These findings indicate that training and supervision may support safe and sustainable integration of peer roles into acute care.

The implementation also faced several challenges. Staff‐related challenges include skepticism about peer roles, unclear expectations, and clinical practices that unintentionally discourage patient‐to‐patient connection.^26^, ^27^, ^28^, ^35^ These results reflect the broader finding that organizational culture and staff attitudes significantly shape the feasibility of peer integration.^45^, ^49^, ^50^ People in peer roles reported emotional strain, boundary ambiguity, and role‐related stress, particularly in high‐acuity contexts.^29^, ^30^ Structural challenges include the lack of dedicated spaces and unit designs that impede social interaction and limit opportunities for peer engagement.^26^, ^29^

Taken together, these findings suggest that peer support may be more effectively implemented when organizations address the cultural, relational, and structural conditions that shape care. Rather than solely depending on individual efforts, embedding peer support within the operational and ethical frameworks of inpatient psychiatry may provide a more stable foundation for long‐term integration.

When interpreted alongside evidence from community and chronic psychiatric contexts, the present findings suggest that peer support in acute inpatient care represents a contextually adapted, rather than fundamentally distinct, form of recovery‐oriented practice. In community and chronic settings, peer workers develop sustained and reciprocal relationships that promote empowerment, social inclusion, and self‐determination, often acting as companions and advocates who help foster hope, autonomy, and a sense of belonging.^14^, ^44^ Conversely, in acute psychiatric inpatient settings, admissions are short and practices are risk‐focused, limiting continuity and shared decision‐making.^1^, ^4^, ^5^ In the present review, even within these constraints, peer supporters were found to provide reassurance, orientation, and relational safety, bridging patients and staff to enhance emotional stability and support the beginning of recovery. These features may represent the distinctive characteristics of acute settings, where peer support focuses on relational and emotional engagement that fosters a sense of safety, stability, and everyday normalcy within the ward environment. Molin et al.^51^ further demonstrated that in acute inpatient care, the quality rather than the quantity of interactions determines patients' sense of safety, dignity, and normalcy. From this perspective, even brief but genuine peer encounters may hold particular value in acute settings, as they can foster mutual understanding and contribute to restoring a sense of personal agency and hope. Peer support in acute settings may have the potential to foster connectedness and hope, reflecting key recovery‐oriented values even within the constraints of acute psychiatric care.

### Stakeholder reflections

Stakeholder interviews with occupational therapists and peer supporters confirmed the already highlighted findings. One occupational therapist remarked that the results aligned with their expectations and described a recent experience with piloting peer support in an acute psychiatric ward. They emphasized the importance of role clarity and coordination, noting that without these, peer supporters risk becoming marginalized. The presence of multiple peer workers has been suggested to mitigate this problem.

Peer supporters reported uncertainty about their expected level of involvement, such as whether they should attend conferences or consult nearby staff. They also expressed concerns about being caught by patients and clinical staff, which they described as emotionally burdensome. The need for peer‐to‐peer support among peer workers was also mentioned.

One peer supporter noted that outside‐structured programs and informal interactions among patients were limited, partly because of staff concerns. In their experience in an acute psychiatric ward, forming close bonds could lead to emotional distress upon a peer's discharge, which resulted in the postponement of their own discharge. Therefore, they felt that limiting peer‐to‐peer interactions may be unavoidable in acute settings. While a peer supporter shared that group sessions sometimes included emotionally meaningful moments, a clinical staff member later indicated the importance and preference for less emotional topics. One occupational therapist commented that such emotional sharing might promote self‐disclosure; however, clinical priorities often make this difficult to achieve in acute settings.

Concerns were raised regarding perceived inequalities in employment arrangements. Some peer supporters preferred external dispatch over in‐house employment because working within the hospital was perceived as possibly compromising the principle of equality. There was also a concern that peer workers might be viewed as occupying a lower position within the hospital hierarchy.

### Strengths and limitations

To our knowledge, this scoping review is the first to comprehensively synthesize and contextualize evidence on peer support, specifically in acute psychiatric inpatient settings. The inclusion of stakeholder reflections from occupational therapists and peer supporters who are actively engaged in peer support practices, including acute settings, adds important practice‐based insights and enhances the clinical relevance of the findings.

This review has several limitations. First, the included studies varied in design, scope, and contextual details, limiting comparisons across settings and precluding the synthesis of effectiveness. Second, this review focused specifically on inpatient settings and did not examine peer support in transitional or community reintegration programs. Therefore, these findings may not reflect the full continuum of peer involvement in service settings. Finally, because this scoping review aimed to map existing evidence rather than evaluate intervention effectiveness, no risk‐of‐bias assessment was conducted. This is consistent with the scoping review methodology; however, it limits the ability to assess the quality or strength of the evidence. Moreover, the limited number of studies and the predominance of qualitative and descriptive designs further restrict the ability to assess effectiveness and generalizability. These limitations highlight the need for further rigorous controlled studies to strengthen the evidence based on peer support in acute psychiatric inpatient settings.

## CONCLUSIONS

This review synthesizes diverse models of peer support in acute psychiatric inpatient care and highlights their contributions to recovery‐oriented practices. While peer support offers emotional, practical, and relational value, its implementation is shaped by organizational culture, staff attitudes, and structural constraints. To realize its potential, future efforts should focus on role clarity, supportive supervision, and organizational structures that enable integration, while preserving the distinct value of peer involvement in high‐acuity settings. With appropriate support and contextual adaptation, peer support has the potential to enhance inpatient experiences and promote recovery, even in acute psychiatric settings.

## AUTHOR CONTRIBUTIONS

Michio Maruta, Goro Tanaka, and Akira Imamura conceptualized and designed the study. Toshio Higashi was responsible for methodology and supervision. Michio Maruta and Goro Tanaka conducted the project administration. Article selection, review, and data curation were performed by Michio Maruta, Gwanghee Han, Kikuo Eguchi, Daiki Yamazono, Goro Tanaka, Tomofusa Kawano, Yuri Onomichi, and Miki Yamashita contributed to the interpretation of the findings. Michio Maruta and Suguru Shimokihara prepared original drafts of the manuscript. All authors reviewed and approved the final version of the manuscript.

## CONFLICT OF INTEREST STATEMENT

The authors declare no conflicts of interest.

## ETHICS APPROVAL STATEMENT

N/A.

## PATIENT CONSENT STATEMENT

N/A.

## CLINICAL TRIAL REGISTRATION

N/A.

## Supporting information

Supp Material.

## Data Availability

All relevant data are available in the manuscript and Supplementary Materials. All data relevant to the study are included in the article or uploaded as supplementary information.
