# Supplementary material for: Peer support in acute psychiatric inpatient settings: A scoping review
Source: PCN Rep. 2025 Nov 14;4(4):e70248. doi: 10.1002/pcn5.70248 (PMC12616875; doi:10.1002/pcn5.70248)
Supplement: Supplementary file 1 — Supp Material. [file PCN5-4-e70248-s001.pdf]

Supplemental Material

Table S1. Search Strategies

Database	No.	Search Term	Results
PubMed	#1	"Peer Group"[MeSH] OR "Self-Help Groups"[MeSH]	36,348
	#2	"peer support*" OR "peer service*" OR "peer program*" OR "peer work*" OR "peer intervention*" OR "peer provi*" OR "peer group*" OR "peer organi*" OR "peer network*" OR "peer initiat*" OR "peer specialis*" OR "peer counsel*" OR "peer approach*" OR "peer advoca*" OR "peer consult*" OR "peer run*" OR "peer based" OR "peer delivered" OR "peer facilitated" OR "peer assist*" OR "peer-led" OR "peer staff*" OR "peer-to-peer" OR "consumer led" OR "consumer run service*" OR "consumer run" OR "consumer based" OR "consumer driven*" OR "consumer directed" OR "consumer delivered" OR "consumer operated" OR "consumer service*" OR "consumer group*" OR "consumer program*" OR "consumer organi*" OR "consumer intervention*" OR "consumer network*" OR "consumer care*" OR "consumer treatment*" OR "consumer provi*" OR "consumer approach*" OR "consumer advoca*" OR "consumer initiat*" OR "consumer consult*" OR "consumer counsel*" OR "consumer work*"	22,687
	#3	#1 OR #2	52,888
	#4	"Mental Health Services"[MeSH] OR "Mental Health Associations"[MeSH] OR "Hospitals, Psychiatric"[MeSH] OR "Psychiatric Department, Hospital"[MeSH]	137,661
	#5	"mental health clinic*" OR "mental health hospital*" OR "mental health facilit*" OR "psychiatric hospital*" OR "psychiatric clinic*" OR “psychiatric unit*” OR "psychiatric ward*” OR “behavioral health hospital*" OR "mental health setting" OR "psychiatr* setting"	28,235
	#6	#4 OR #5	154,185
	#7	Inpatients [MeSH] OR Hospitalization [MeSH]	334,401
	#8	"inpatient*" OR "in-patient*" OR "hospitalised" OR "hospitalized" OR "admitted" OR "hospital stay"	2,788,424
	#9	#7 OR #8	2,942,263
	#10	[MeSH] "Subacute Care"	1,502
	#11	"Subacute Care" OR "acute care*" OR acute OR subacute OR postacute OR sub-acute OR post-acute OR "acute phase*" OR "subacute phase*" OR "sub-acute phase*" OR "postacute phase*" OR "post-acute phase*" OR "early phase*" OR "initial phase*"	1,622,939

	#12	#10 OR #11	1,623,332
	#13	#3 AND #6 AND #9 AND #12	48
Scopus	#1	"peer support*" OR "peer service*" OR "peer program*" OR "peer work*" OR "peer intervention*" OR "peer provi*" OR "peer group*" OR "peer organi*" OR "peer network*" OR "peer initiat*" OR "peer specialis*" OR "peer counsel*" OR "peer approach*" OR "peer advoca*" OR "peer consult*" OR "peer run*" OR "peer based" OR "peer delivered" OR "peer facilitated" OR "peer assist*" OR "peer-led" OR "peer staff*" OR "peer-to-peer" OR "consumer led" OR "consumer run service*" OR "consumer run" OR "consumer based" OR "consumer driven*" OR "consumer directed" OR "consumer delivered" OR "consumer operated" OR "consumer service*" OR "consumer group*" OR "consumer program*" OR "consumer organi*" OR "consumer intervention*" OR "consumer network*" OR "consumer care*" OR "consumer treatment*" OR "consumer provi*" OR "consumer approach*" OR "consumer advoca*" OR "consumer initiat*" OR "consumer consult*" OR "consumer counsel*" OR "consumer work*" OR "self-help" OR "self-help group*"	156,795
	#2	"mental health clinic*" OR "mental health hospital*" OR "mental health facilit*" OR "psychiatric hospital*" OR "psychiatric clinic*" OR “psychiatric unit*” OR "psychiatric ward*” OR "behavioral health hospital*" OR "mental health setting" OR "psychiatr* setting"	42,529
	#3	"inpatient*" OR "in-patient*" OR "hospitalised" OR "hospitalized" OR "admitted" OR "hospital stay" OR "hospitalization"	3,556,105
	#4	"Subacute Care" OR "acute care*" OR acute OR subacute OR postacute OR sub-acute OR post-acute OR "acute phase*" OR "subacute phase*" OR "sub-acute phase*" OR "postacute phase*" OR "post-acute phase*" OR "early phase*" OR "initial phase*"	2,329,181
	#5	#1 AND #2 AND #3 AND #4	43
MEDLINE	S1	"Peer Group"[MeSH] OR "Self-Help Groups"[MeSH]	34,543
	S2	"peer support*" OR "peer service*" OR "peer program*" OR "peer work*" OR "peer intervention*" OR "peer provi*" OR "peer group*" OR "peer organi*" OR "peer network*" OR "peer initiat*" OR "peer specialis*" OR "peer counsel*" OR "peer approach*" OR "peer advoca*" OR "peer consult*" OR "peer run*" OR "peer based" OR "peer delivered" OR "peer facilitated" OR "peer assist*" OR "peer-led" OR "peer staff*" OR "peer-to-peer" OR "consumer led" OR "consumer run service*" OR "consumer run" OR "consumer based" OR "consumer driven*" OR "consumer directed" OR "consumer delivered" OR "consumer operated" OR "consumer service*" OR "consumer group*" OR "consumer	21,243

		program*" OR "consumer organi*" OR "consumer intervention*" OR "consumer network*" OR "consumer care*" OR "consumer treatment*" OR "consumer provi*" OR "consumer approach*" OR "consumer advoca*" OR "consumer initiat*" OR "consumer consult*" OR "consumer counsel*" OR "consumer work*" OR "self-help" OR "self-help group*"	
	S3	S1 OR S2	50,035
	S4	"Mental Health Services"[MeSH] OR "Mental Health Associations"[MeSH] OR "Hospitals, Psychiatric"[MeSH] OR "Psychiatric Department, Hospital"[MeSH]	88,309
	S5	"mental health clinic*" OR "mental health hospital*" OR "mental health facilit*" OR "psychiatric hospital*" OR "psychiatric clinic*" OR “psychiatric unit*” OR "psychiatric ward*” OR “behavioral health hospital*" OR "mental health setting" OR "psychiatr* setting"	25,108
	S6	S4 OR S5	102,872
	S7	Inpatients [MeSH] OR Hospitalization [MeSH]	173,533
	S8	"inpatient*" OR "in-patient*" OR "hospitalised" OR "hospitalized" OR "admitted" OR "hospital stay"	2,772,958
	S9	S7 OR S8	2,844,840
	S10	[MeSH] "Subacute Care"	1,502
	S11	"Subacute Care" OR "acute care*" OR acute OR subacute OR postacute OR sub-acute OR post-acute OR "acute phase*" OR "subacute phase*" OR "sub-acute phase*" OR "postacute phase*" OR "post-acute phase*" OR "early phase*" OR "initial phase*"	1,590,849
	S12	S10 OR S11	1,591,245
	S13	S3 AND S6 AND S9 AND S12	39
CHINAHL	S1	[Heading] "Peer Group" OR "Support Groups"	28,831
	S2	"peer support*" OR "peer service*" OR "peer program*" OR "peer work*" OR "peer intervention*" OR "peer provi*" OR "peer group*" OR "peer organi*" OR "peer network*" OR "peer initiat*" OR "peer specialis*" OR "peer counsel*" OR "peer approach*" OR "peer advoca*" OR "peer consult*" OR "peer run*" OR "peer based" OR "peer delivered" OR "peer facilitated" OR "peer assist*" OR "peer-led" OR "peer staff*" OR "peer-to-peer" OR "consumer led" OR "consumer run service*" OR "consumer run" OR "consumer based" OR "consumer driven*" OR "consumer directed" OR "consumer delivered" OR "consumer operated" OR "consumer service*" OR "consumer group*" OR "consumer program*" OR "consumer organi*" OR "consumer intervention*" OR "consumer network*" OR "consumer care*" OR "consumer treatment*" OR "consumer provi*" OR "consumer approach*" OR "consumer advoca*" OR "consumer	14,300

		initiat*" OR "consumer consult*" OR "consumer counsel*" OR "consumer work*"	
	S3	S1 OR S2	38,338
	S4	[Heading] "Mental Health Services" OR "Mental Health Organizations" OR "Hospitals, Psychiatric" OR "Psychiatric Units" OR "Psychiatric Service"	51,298
	S5	"mental health clinic*" OR "mental health hospital*" OR "mental health facilit*" OR "psychiatric hospital*" OR "psychiatric clinic*" OR "psychiatric unit*" OR "psychiatric ward*" OR "behavioral health hospital*" OR "mental health setting" OR "psychiatr* setting"	11,796
	S6	S4 OR S5	58,245
	S7	[Heading] Inpatients OR Hospitalization	136,072
	S8	"inpatient*" OR "in-patient*" OR "hospitalised" OR "hospitalized" OR "admitted" OR "hospital stay"	706,753
	S9	S7 OR S8	784,349
	S10	[Heading] "Acute Care" OR "Subacute Care"	13,069
	S11	"Subacute Care" OR "acute care*" OR acute OR subacute OR postacute OR sub-acute OR post-acute OR "acute phase*" OR "subacute phase*" OR "sub-acute phase*" OR "postacute phase*" OR "post-acute phase*" OR "early phase*" OR "initial phase*"	329,266
	S12	S10 OR S11	333,278
	S13	S3 AND S6 AND S9 AND S12	33
ProQuest	S1	"peer support*" OR "peer service*" OR "peer program*" OR "peer work*" OR "peer intervention*" OR "peer provi*" OR "peer group*" OR "peer organi*" OR "peer network*" OR "peer initiat*" OR "peer specialis*" OR "peer counsel*" OR "peer approach*" OR "peer advoca*" OR "peer consult*" OR "peer run*" OR "peer based" OR "peer delivered" OR "peer facilitated" OR "peer assist*" OR "peer-led" OR "peer staff*" OR "peer-to-peer" OR "consumer led" OR "consumer run service*" OR "consumer run" OR "consumer based" OR "consumer driven*" OR "consumer directed" OR "consumer delivered" OR "consumer operated" OR "consumer service*" OR "consumer group*" OR "consumer program*" OR "consumer organi*" OR "consumer intervention*" OR "consumer network*" OR "consumer care*" OR "consumer treatment*" OR "consumer provi*" OR "consumer approach*" OR "consumer advoca*" OR "consumer initiat*" OR "consumer consult*" OR "consumer counsel*" OR "consumer work*" OR "self-help" OR "self-help group*"	65,197
	S2	"mental health clinic*" OR "mental health hospital*" OR "mental health facilit*" OR "psychiatric hospital*" OR "psychiatric clinic*" OR "psychiatric unit*" OR "psychiatric ward*" OR "behavioral health hospital*" OR "mental	40,311

	health setting" OR "psychiatr* setting"	
S3	"inpatient*" OR "in-patient*" OR "hospitalised" OR "hospitalized" OR "admitted" OR "hospital stay" OR "hospitalization"	4,307,706
S4	"Subacute Care" OR "acute care*" OR acute OR subacute OR postacute OR sub-acute OR post-acute OR "acute phase*" OR "subacute phase*" OR "sub-acute phase*" OR "postacute phase*" OR "post-acute phase*" OR "early phase*" OR "initial phase*"	2,434,391
S5	S1 AND S2 AND S3 AND S4	48
